# Exploring the frontiers of metal additive manufacturing in orthopaedic implant development

**DOI:** 10.1016/j.mex.2024.103056

**Published:** 2024-11-15

**Authors:** Senthil Maharaj Kennedy, Vasanthanathan A, Amudhan K

**Affiliations:** a Department of Mechanical Engineering, AAA College of Engineering and Technology, Sivakasi 626005, Tamil Nadu, India; b Department of Mechanical Engineering, Mepco Schlenk Engineering College, Sivakasi 626005, Tamil Nadu, India

**Keywords:** Fabrication and Testing methods in Metal Additive Manufacturing for Orthopedic Implant Development, Additive manufacturing, Metals, Implant, Titanium, Biocompatibility

## Abstract

This paper provides a thorough analysis of recent advancements and emerging trends in the integration of metal additive manufacturing (AM) within orthopedic implant development. With an emphasis on the use of various metals and alloys, including titanium, cobalt-chromium, and nickel-titanium, the review looks at their characteristics and how they relate to the creation of various orthopedic implants, such as spinal implants, hip and knee replacements, and cranial-facial reconstructions. The study highlights how metal additive manufacturing (AM) can revolutionize the field by enabling customized implant designs that take patient anatomical variances into account. The review discusses the drawbacks of conventional manufacturing techniques and emphasizes the benefits of metal additive manufacturing (AM), such as increased design flexibility and decreased material waste. Important material selection factors, including mechanical qualities and biocompatibility, are covered in relation to metal additive manufacturing applications. The work ends with a summary of the issues facing metal AM today, such as surface finish and material certification, and suggestions for future developments, like the creation of advanced materials and the application of AI to design optimization.

## Review Highlights

• Highlights the advances in metal AM for orthopedics and the role of metal additive manufacturing (AM) in enabling custom, patient-specific orthopedic implants.
• Explores material and process innovations in biocompatible metals and advanced AM techniques to improve implant performance and durability.
• Discusses current challenges in metal AM, including quality control and material certification, and outlines future research opportunities for the field.

## Graphical abstract

**Figure:**
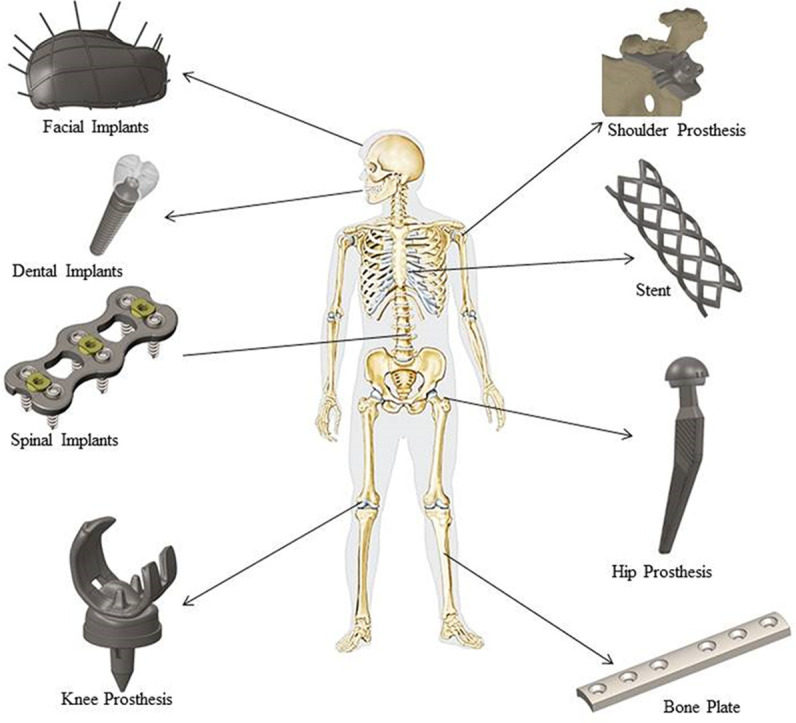

Specifications table

**Table:** 

Subject area:	Engineering
More specific subject area:	Additive Manufacturing
Name of the reviewed methodology:	Fabrication and Testing methods in Metal Additive Manufacturing for Orthopedic Implant Development
Keywords:	CFRP composite, filament winding, pultrusion, mechanical characterization, non-destructive testing
Resource availability:	• ingh R, Gupta A, Tripathi O, Srivastava S, Singh B, Awasthi A, et al. Powder bed fusion process in additive manufacturing: An overview. Mater Today Proc 2020;26:3058–70. https://doi.org/10.1016/J.MATPR.2020.02.635. • Le VD, Pessard E, Morel F, Prigent S. Fatigue behaviour of additively manufactured Ti-6Al-4 V alloy: The role of defects on scatter and statistical size effect. Int J Fatigue 2020;140:105,811. https://doi.org/10.1016/J.IJFATIGUE.2020.105811. • Ahmadi SM, Hedayati R, Li Y, Lietaert K, Tümer N, Fatemi A, et al. Fatigue performance of additively manufactured meta-biomaterials: The effects of topology and material type. Acta Biomater 2018;65:292–304. https://doi.org/10.1016/J.ACTBIO.2017.11.014. • Ahn DG. Directed Energy Deposition (DED) Process: State of the Art. Int J Precis Eng Manuf Technol 2021 82 2021;8:703–42. https://doi.org/10.1007/S40684–020–00302–7. • Svetlizky D, Das M, Zheng B, Vyatskikh AL, Bose S, Bandyopadhyay A, et al. Directed energy deposition (DED) additive manufacturing: Physical characteristics, defects, challenges and applications. Mater Today 2021;49:271–95. https://doi.org/10.1016/J.MATTOD.2021.03.020. • Lim JS, Oh WJ, Lee CM, Kim DH. Selection of effective manufacturing conditions for directed energy deposition process using machine learning methods. Sci Rep 2021;11:1–13. https://doi.org/10.1038/s41598–021–03622-z. • Li SH, Kumar P, Chandra S, Ramamurty U. Directed energy deposition of metals: processing, microstructures, and mechanical properties. Int Mater Rev 2023;68:605–47. https://doi.org/10.1080/09506608.2022.2097411. • Zhou Z, Lennon A, Buchanan F, McCarthy HO, Dunne N. Binder jetting additive manufacturing of hydroxyapatite powders: Effects of adhesives on geometrical accuracy and green compressive strength. Addit Manuf 2020;36:101,645. https://doi.org/10.1016/J.ADDMA.2020.101645.
Review question:	1. What are the key advantages of using metal additive manufacturing (AM) in orthopedic implant development? 2. Which metals are commonly used in metal AM for orthopedic implants, and why are they preferred? 3. How does metal AM improve the design and customization of orthopedic implants compared to traditional manufacturing methods? 4. What are the main challenges faced in using metal AM for orthopedic implants? 5. What future research directions are proposed to overcome the limitations of metal AM in orthopedic implant development?

## Background

In the development of orthopaedic implants, metal additive manufacturing is the process of creating customised implants for orthopaedic use by utilising cutting-edge 3D printing technologies and metal materials [1,2]. Orthopaedic implants have traditionally been produced via subtractive manufacturing, which involves removing material from a solid block. Metal AM, on the other hand, constructs the implant layer by layer, allowing for complex and patient-specific designs. With the use of this creative method, design flexibility is increased and implants can be customised to fit each patient's specific anatomical features [3,4]. Moreover, metal additive manufacturing (AM) in orthopaedic implant development makes it easier to create complex structures with optimal biomechanical properties, which may improve implant longevity and performance. Furthermore, by using this technique, implants with porous structures can be created, which would improve osseointegration and lower the likelihood of implant rejection. All things considered, metal additive manufacturing transforms the field of orthopaedic implant development by providing a more customised, accurate, and adaptable manufacturing process, which has the potential to enhance patient outcomes and spur medical innovation [5,6].

There are major ramifications for the orthopedic implant development field from investigating the limits of metal additive manufacturing (AM). Based on unique anatomical information, Metal AM enables the development of orthopedic implants tailored to each patient [[7], [8], [9], [10]]. Customization has the potential to enhance patient outcomes, shorten recovery times, and increase overall satisfaction by optimizing the fit and functionality of implants [11,12]. Complex and intricate structures that were previously difficult or impossible to produce using conventional manufacturing methods can now be fabricated thanks to technology. With improved biomechanical qualities, implants can be designed with greater potential for minimizing complications and better integration with the surrounding tissues. A range of cutting-edge materials, including alloys with particular mechanical qualities appropriate for orthopedic applications, can be used with metal additive manufacturing (AM). This removes some of the constraints associated with using traditional materials and creates opportunities for the development of implants with increased strength, durability, and biocompatibility [13,14]. Metal additive manufacturing (AM) creates things layer by layer, in contrast to traditional subtractive manufacturing, where extra material is frequently thrown away. This can lead to less wasteful use of materials, improving the sustainability and environmental friendliness of the manufacturing process. Rapid prototyping made possible by metal additive manufacturing (AM) enables designers and producers to quickly iterate and improve designs. This quickens the development cycle, which facilitates the incorporation of fresh ideas into implant design and the response to new challenges as they arise. Complex fractures or deformities are examples of orthopedic cases that may call for highly customized care. With its ability to handle complex cases with custom implant designs, Metal AM may be able to improve surgical outcomes in difficult situations. Researchers can push the limits of what is currently feasible in orthopedic implant development by delving into the frontiers of metal AM. By encouraging creativity and adding to the on-going development of orthopedic procedures, this research opens the door for future discoveries and innovations. Technology is a promising tool for improving orthopedic medicine because it can solve complex problems and provide individualized solutions [[15], [16], [17]].

## Method details

### Metal additive manufacturing in orthopedic implant development

Metal additive manufacturing (AM) encompasses a range of techniques that utilize layer-by-layer deposition of metal materials to create three-dimensional objects. These techniques offer unprecedented design flexibility, allowing for complex geometries and customized structures.

#### Powder bed fusion (PBF)

**Fig. 1:**
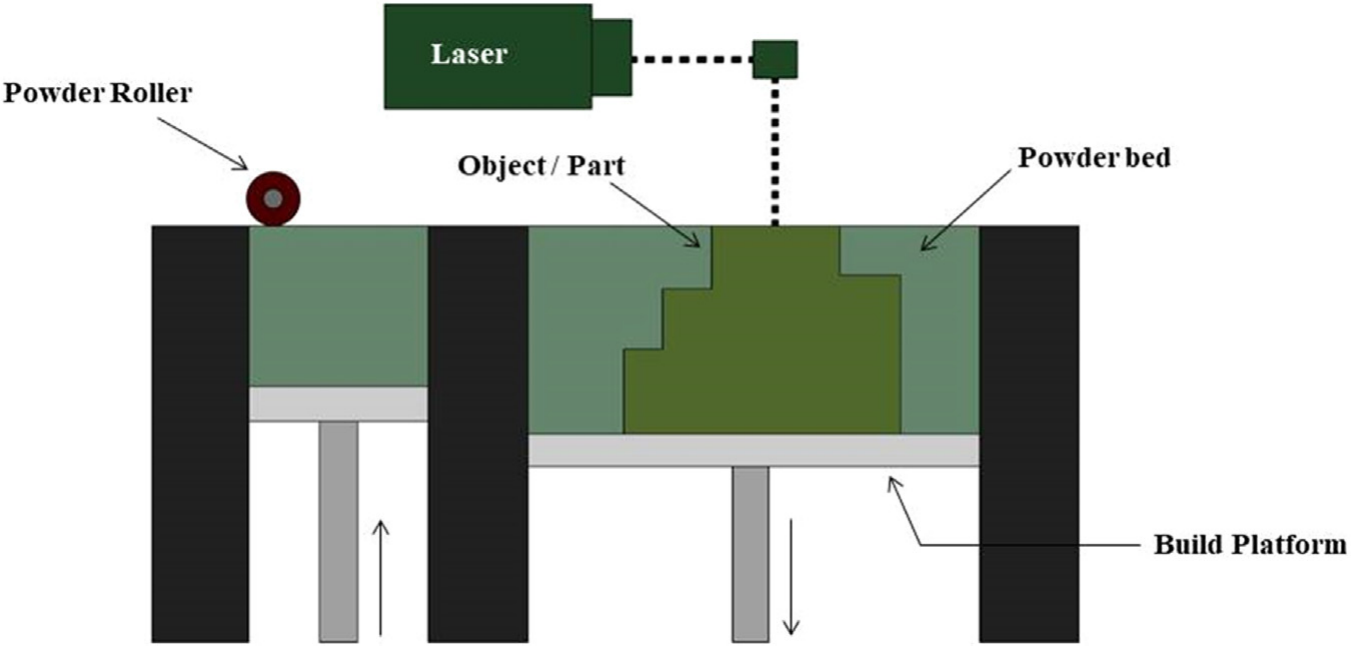
Powder Bed Fusion process.

In order to create three-dimensional objects, a type of metal additive manufacturing (AM) process called powder bed fusion (Fig. 1) involves selectively melting layers of metal powder. In order to prevent oxidation, the process is usually carried out in a controlled chamber with an inert gas atmosphere. PBF techniques like Electron Beam Melting (EBM) and Selective Laser Melting (SLM) have become more popular because of their exceptional precision in producing complex and fully dense metal components [18,19]. The entire build platform is covered in a thin layer of metal powder. Usually, the layer thickness falls between 20 and 100 micrometers. The metal powder is selectively melted using a high-energy source, such as an electron beam (EBM) or a laser (SLM), in accordance with the digital design data. The build platform is lowered and a fresh layer of metal powder is applied on top of the previously melted layer. Layer by layer, the procedure is repeated until the entire object is created. The metal quickly cools and solidifies as it melts, combining with the layers that came before it. This guarantees a completely uniform and dense structure. Following printing, the created object goes through post-processing procedures. This could entail doing heat treatments and eliminating extra powder [20,21].

#### Directed energy deposition (DED)

**Fig. 2:**
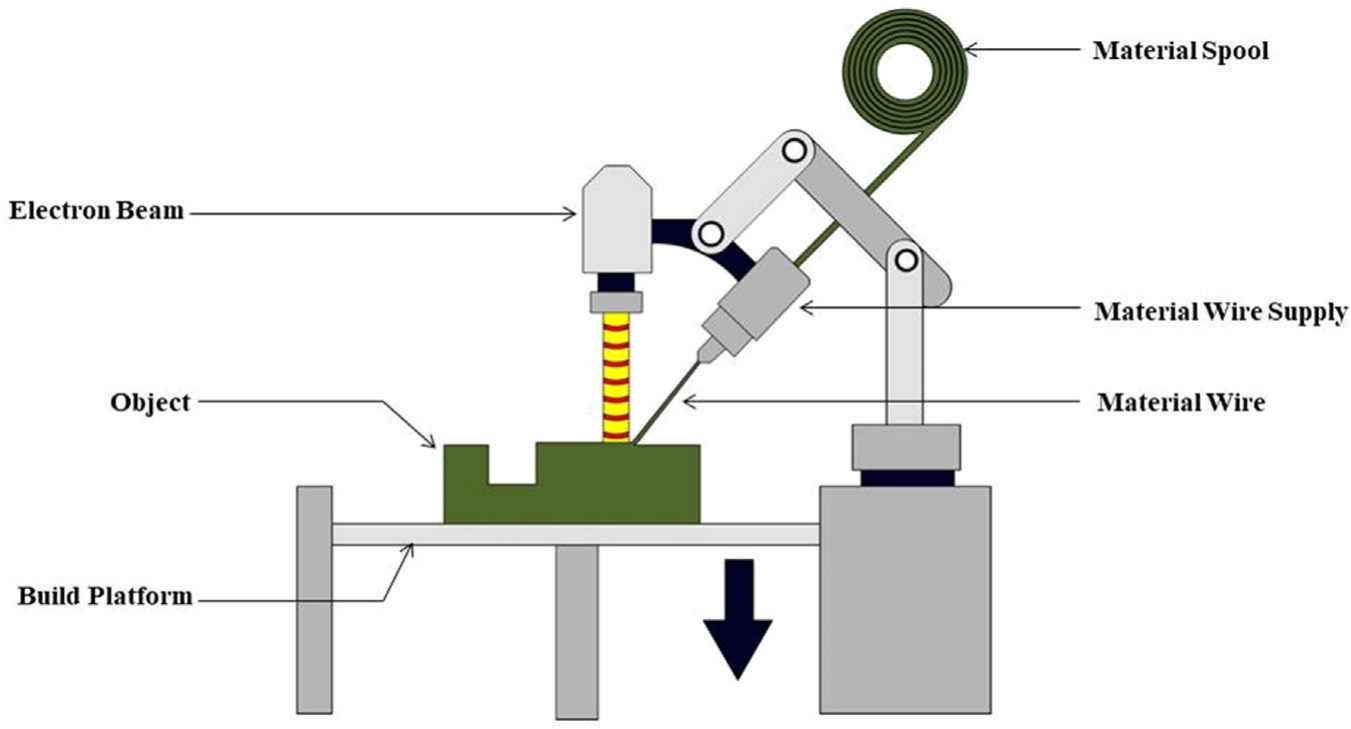
Directed Energy Deposition process.

A focused energy source, like a laser or electron beam, is used in the directed energy deposition (DED) method of metal additive manufacturing (AM) to precisely deposit metal wire or powder. DED is different from other AM methods in that it continuously melts and deposits material to build the object layer by layer. Because of its adaptability, this process can be used for both additive manufacturing and repair applications [[22], [23], [24]]. By means of a nozzle or another delivery device, metal powder or wire is introduced into the deposition zone. As the material is deposited, the focused energy source—a laser or electron beam—melts it. Layer by layer, the desired geometry is constructed by precisely depositing the melted material onto the substrate or the preceding layer. Large and complex components can be created because the process is continuous. A metallurgical bonded layer is formed as the material is deposited, melts, and quickly cools and solidifies. DED systems frequently feature multi-axis robotic arms or other mechanisms that allow for the fabrication of intricate three-dimensional shapes by offering flexibility in the deposition process. Certain DED systems have real-time control and monitoring capabilities to guarantee the calibre of the deposition procedure. This may involve sensors that monitor temperature, melt pool characteristics, and other parameters [25,26] (Fig. 2).

#### Binder jetting

**Fig. 3:**
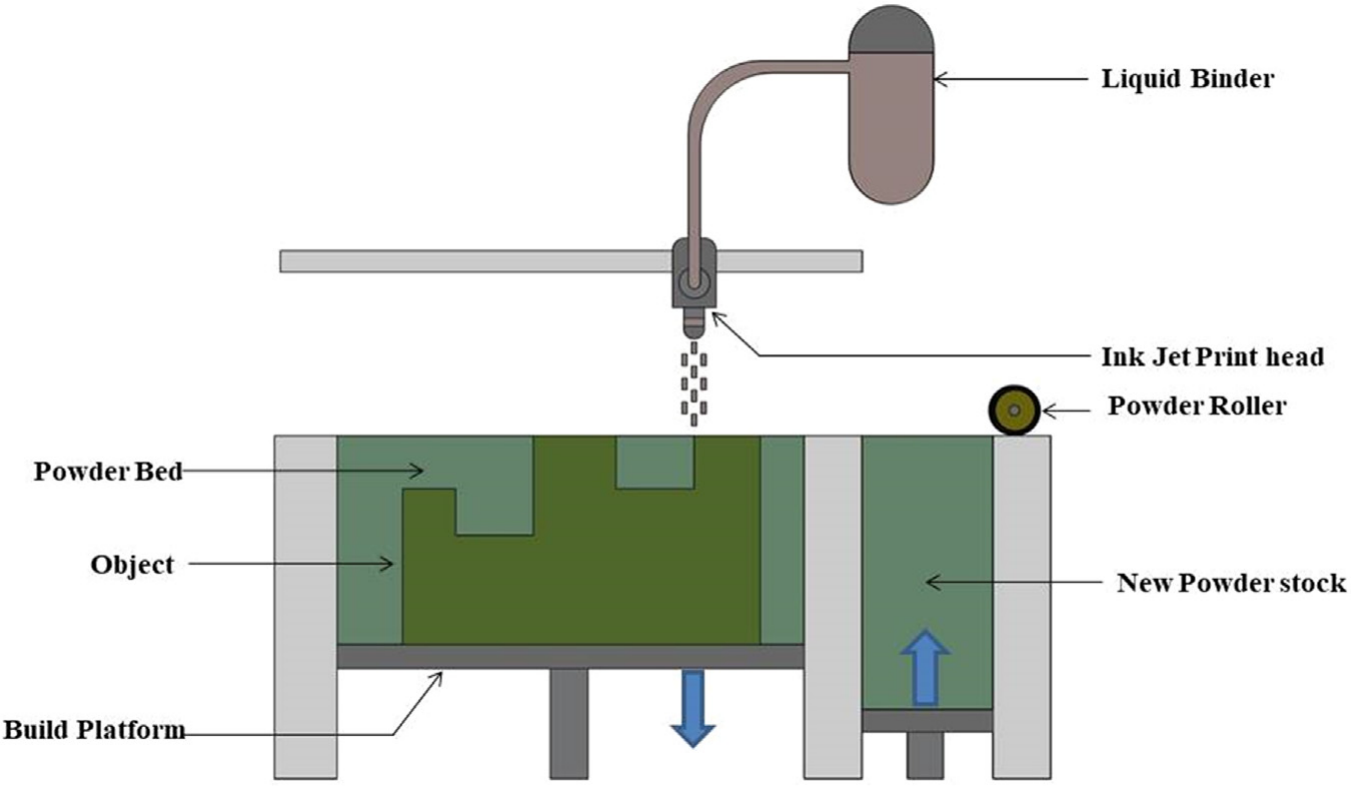
Binder jetting process.

An additive manufacturing (AM) process called "binder jetting" (Fig. 3) entails carefully applying a liquid binding agent to a layer of metal powder, joining the particles to form a three-dimensional object. The process is repeated until the entire object is formed, spreading a fresh layer of metal powder after each layer is deposited [27,28]. To obtain the final metal component, the green portion is then frequently sintered or subjected to additional processing. Although binder jetting is extensively utilised in the production of polymers and ceramics, its application to metals has drawn interest due to its ability to produce complex parts through scalable and affordable manufacturing [29,30]. Metal powder is evenly distributed in a thin layer across the build platform. In accordance with the digital design data, a liquid binding agent is selectively deposited onto the powder layer to bind the metal powder particles together in the desired pattern. Layer by layer, the binder and powder are selectively deposited during the process until the entire object is formed [31,32]. The loosely bound metal powder, or the "green part," goes through post-processing procedures following binder jetting. Typically, this entails applying heat treatments, like sintering, to attain densification and the desired mechanical characteristics [33,34] (Table 1).

**Table 1:** Pros and Cons of Metal Additive Manufacturing methods.

Metal Additive Manufacturing methods	Advantages	Applications
Powder bed fusion (PBF)	Manufacturing Complex Geometries High Precision Material Variety Customization	Aerospace Medical Automotive Tooling and Prototyping
Directed energy deposition (DED)	Versatility Rapid Build Rates Repair and Cladding Near-Net Shape Manufacturing	Aerospace Automotive Energy Tooling and Moulds
Binder jetting	Speed and Scalability Cost-Effectiveness Complex Geometries Material Variety	Prototyping Large-Scale Production Tooling and Jigs Aerospace and Automotive

### Materials and optimization methodology in metal AM for orthopedic implants

When developing orthopedic implants, choosing the right materials for metal additive manufacturing (AM) is essential because the materials used have a big impact on the mechanical characteristics, biocompatibility, and overall performance of the implant [35,36]. To guarantee that the implant does not cause negative reactions or rejection within the patient's body, the materials chosen must be biocompatible. Biocompatible materials lessen the possibility of implant-related complications or inflammation while promoting successful osseointegration. The body places different mechanical loads and stresses on orthopedic implants. For the materials to withstand these forces and offer long-term stability, they must have the right mechanical qualities, such as strength, elasticity, and fatigue resistance [37,38]. The human body exposes implants to bodily fluids, which over time may cause corrosion. High corrosion resistance materials are crucial for maintaining the longevity and robustness of orthopedic implants, averting deterioration and possible failure. For post-implantation monitoring and diagnostics, radiopacity is essential. Properly radiopacity materials enable distinct visibility in medical imaging methods like X-rays, enabling precise evaluation of implant placement and possible problems [39,40]. In order to ensure ease of fabrication and the ability to create intricate and complex designs, the materials chosen should be compatible with the metal additive manufacturing process. Suitability for particular printing parameters and methods is necessary for productive manufacturing. It is well known that a few substances and the alloys they contain can encourage osseointegration, or the integration of the implant with the surrounding bone. Furthermore, by creating a scaffold for bone ingrowth, metal AM's ability to control the material's porosity can improve osseointegration even more [41,42]. The capacity of metal AM to handle a broad variety of materials accounts for its adaptability. Orthopedic implants can be made more customized by selecting materials that can be modified to meet the unique needs of each patient. This will result in a better fit and better clinical results. Materials with the right heat conductivity to replicate the characteristics of natural bone may be advantageous for certain orthopedic applications, such as joint replacements. This guarantees that the implant's thermal interactions with the surrounding tissues are in line with the physiological processes of the body. To guarantee safety and effectiveness, materials chosen for orthopedic implants must pass rigorous testing and adhere to regulatory requirements. In order to receive approval from health authorities and guarantee that the implants fulfill established quality and safety standards, compliance with regulatory requirements is crucial. One important consideration in the development of orthopedic implants is material cost-effectiveness. Achieving a balance between the cost of materials and performance requirements is crucial to the accessibility and economic viability of implants [[43], [44], [45]].

Metal additive manufacturing (AM) for orthopedic implants involves the use of various metals and alloys, each chosen for its specific properties and suitability for the intended application (Table 2).

**Table 2:** Properties and Applications of Metal AM Materials for Orthopedic implants.

Metal AM Materials for Orthopaedic implants	Pros	Cons	Modulus (GPa) and Strength (MPa)	Applications
Titanium Alloys	• Lightweight • High strength • Excellent corrosion resistance • Biocompatible	• High cost • Difficult to machine • Risk of stress shielding	• 110 GPa • 800–950 MPa	• Hip and knee replacements • Spinal implants • Bone plates.
Cobalt-Chromium Alloys	• High strength • Wear resistance • Corrosion resistance	• High modulus, risk of stress shielding • Difficult to process	• 200–230 GPa • 600–1000 MPa	• Hip and knee implants • Dental applications
Stainless Steel Alloys	• Corrosion resistance • Cost-effective • High strength	• Lower biocompatibility than titanium • Susceptible to wear	• 190–210 GPa • 520–680 MPa	• Bone plates • Bone screws • Joint implants
Nickel-Titanium (Ni-Ti) Alloys (Nitinol)	• Shape memory effect • Super elasticity • Biocompatibility	• Expensive • Nickel content may cause allergies	• 28–41 GPa • 895–960 MPa	• Stents
Zirconium Alloys	• Excellent corrosion resistance • Biocompatibility	• Limited research in orthopaedics	• 100 GPa • 380–800 MPa	• Spinal applications
Tantalum	• High biocompatibility • Corrosion resistance	• Very high cost	• 190 GPa • 350–500 MPa	• Acetabular cups and augmentations
Magnesium Alloys	• Lightweight • Biocompatibility	• Limited mechanical strength	• 41–45 GPa • 100–220 MPa	• Orthopedic implants
Copper Alloys	• Good thermal conductivity • Antimicrobial properties	• Poor mechanical strength for load-bearing • Not widely used in high-stress implants	• 120 GPa • 200–350 MPa	• Joint prostheses

The material of choice is determined by a number of factors, including the particular application, load-bearing specifications, biocompatibility, and the material's suitability for metal AM processing. Continuous investigation and progress in material science keep broadening the spectrum of materials accessible for metal additive manufacturing in the creation of orthopedic implants.

### Mechanical properties testing methods of metal AM orthopedic implants

**Fig. 4:**
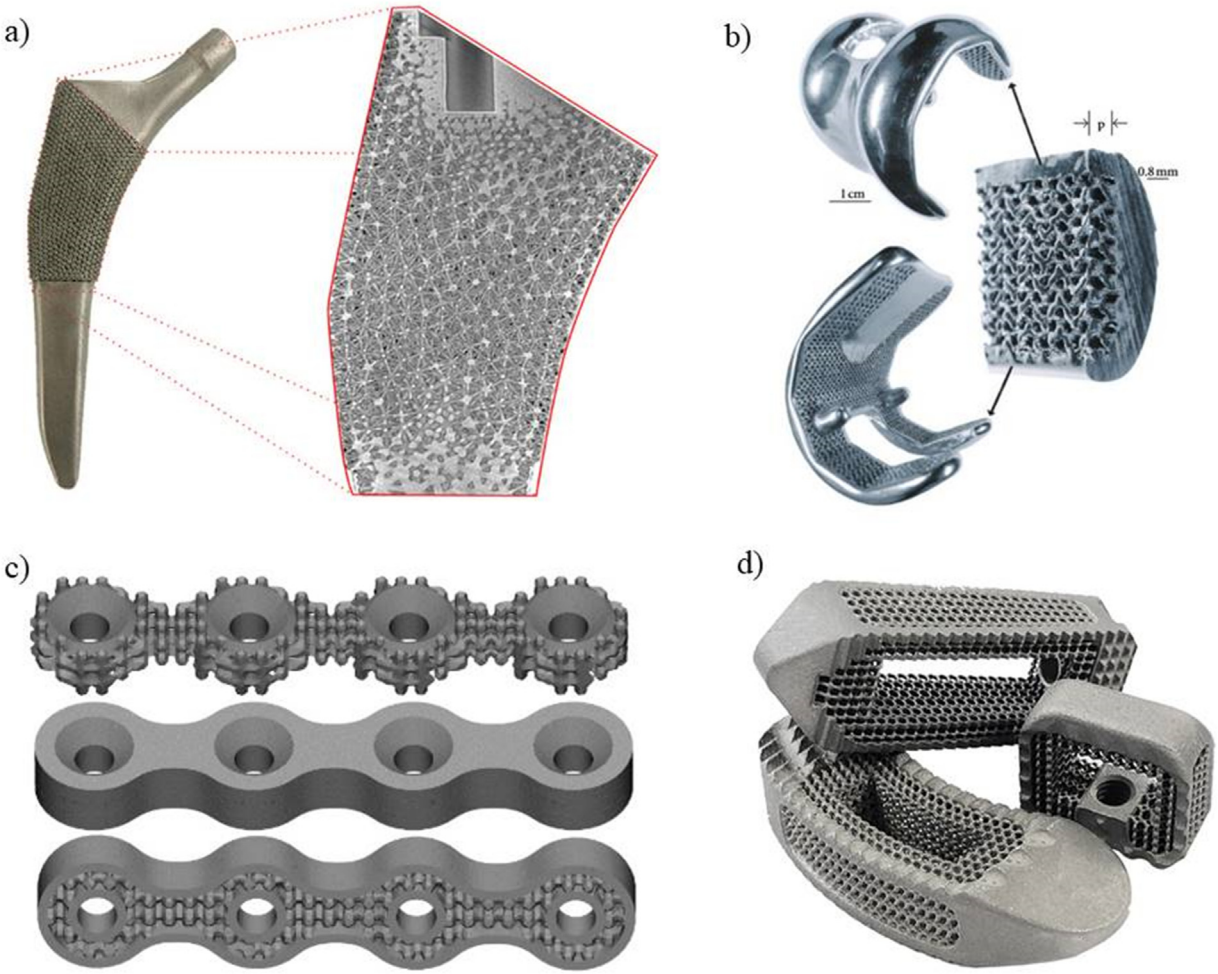
Metal AM implants (a) Hip implant [47] (b) Knee implant [48] (c) Bone plates [49] (d) Spinal implants [50].

As metal additive manufacturing (AM) can create customized implants with complex geometries, it has attracted a lot of interest in the orthopedics implant industry (Fig. 4). Nonetheless, research is being done to examine and contrast the mechanical characteristics of metal AM orthopedic implants with those of implants that are produced traditionally [46].

A variety of materials, such as titanium alloys, cobalt-chromium alloys, and stainless steel, can be used to create metal AM implants. These materials have well-established mechanical characteristics from traditional production methods. However, depending on the particular AM process utilized, the mechanical characteristics and microstructure of metal AM implants may vary [51]. The potential for porosity, which can alter mechanical properties, is an important feature of metal AM implants. Typically, metal additive manufacturing (AM) uses a powder bed fusion method in which the powder particles are melted layer by layer with precision. When compared to implants made using traditional methods, this process has the potential to create porosity and voids in the finished product, which would lower its mechanical strength and fatigue resistance. Density is another issue because layer-by-layer manufacturing of metal AM implants can result in varying densities throughout the structure [[52], [53], [54], [55], [56]].

When compared to implants that are manufactured traditionally, metal AM implants frequently display distinctive microstructural features. Finer grain structures may be produced by the quick cooling and solidification rates in metal AM, which could improve certain mechanical qualities like fatigue resistance. Microstructural flaws, such as incomplete melting, unmelted particles, or non-equilibrium phases, could jeopardize mechanical performance, though [[57], [58], [59], [60]].

**Fig. 5:**
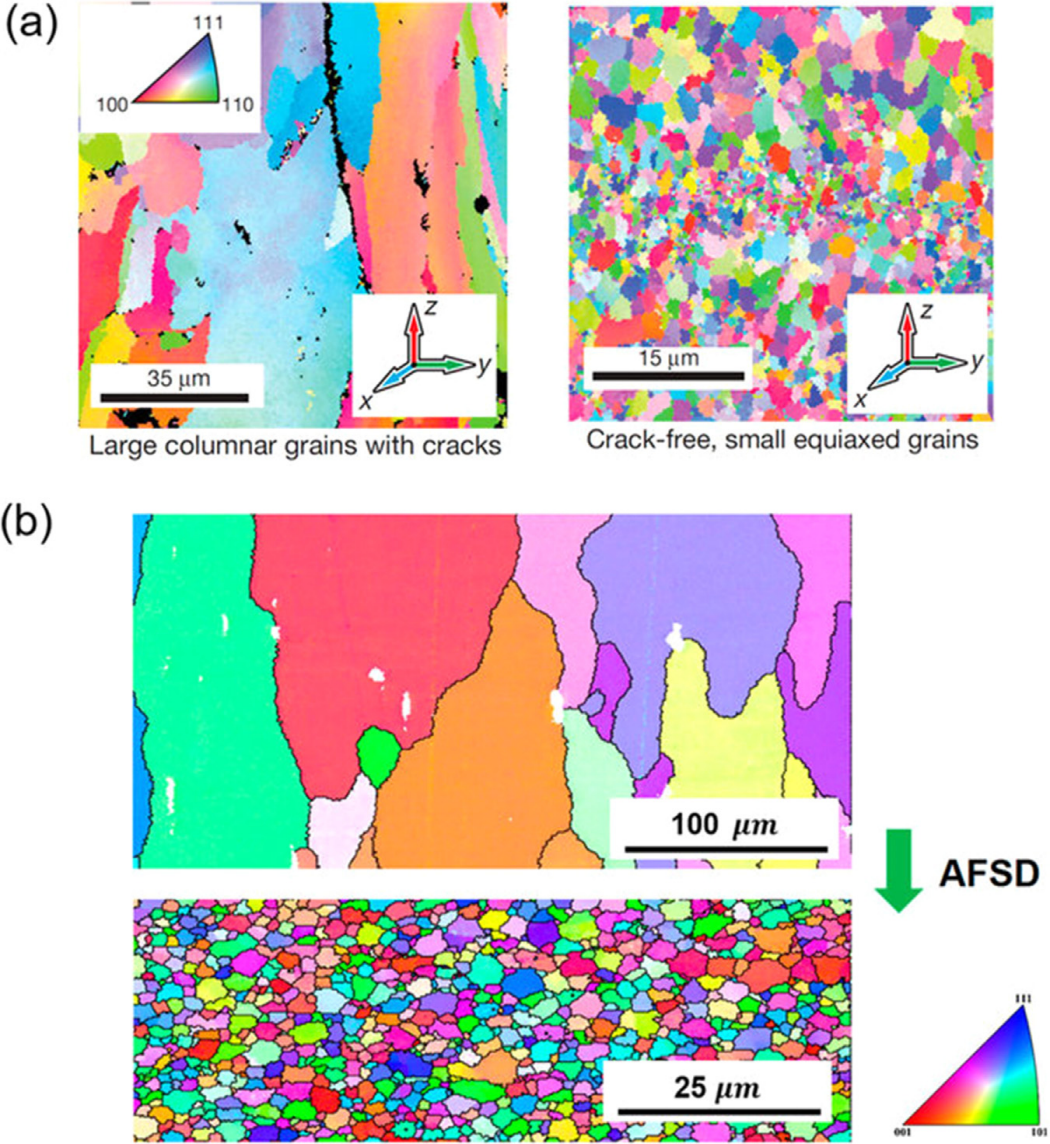
Characteristic microstructures of AA7075 by fusion-based additive manufacturing [63].

Morphology studies of additive manufactured (AM) biomaterials are critical for determining their suitability in clinical applications, as surface characteristics and microstructure have a direct impact on the material's mechanical and biological performance. Surface roughness, porosity, pore interconnectivity, and overall structure are all important factors in determining how well an implant integrates with surrounding tissues and can withstand physiological loads [61]. Surface morphology influences osseointegration, the process by which bone cells adhere to and grow on implants in clinical settings, particularly in orthopedics and dentistry. Research has shown that rougher, micro-textured surfaces promote better bone cell attachment and faster tissue integration. Furthermore, porosity and pore structure in AM biomaterials are carefully designed to promote vascularization, nutrient exchange, and tissue ingrowth, all of which are critical for long-term implant stability. Researchers can assess these morphological aspects and adjust the design parameters by employing advanced imaging techniques such as scanning electron microscopy (SEM) and micro-computed tomography (micro-CT) [62]. Fig. 5 explains the characteristic microstructures of aluminum alloy 7075 by fusion-based additive manufacturing.

**Fig. 6:**
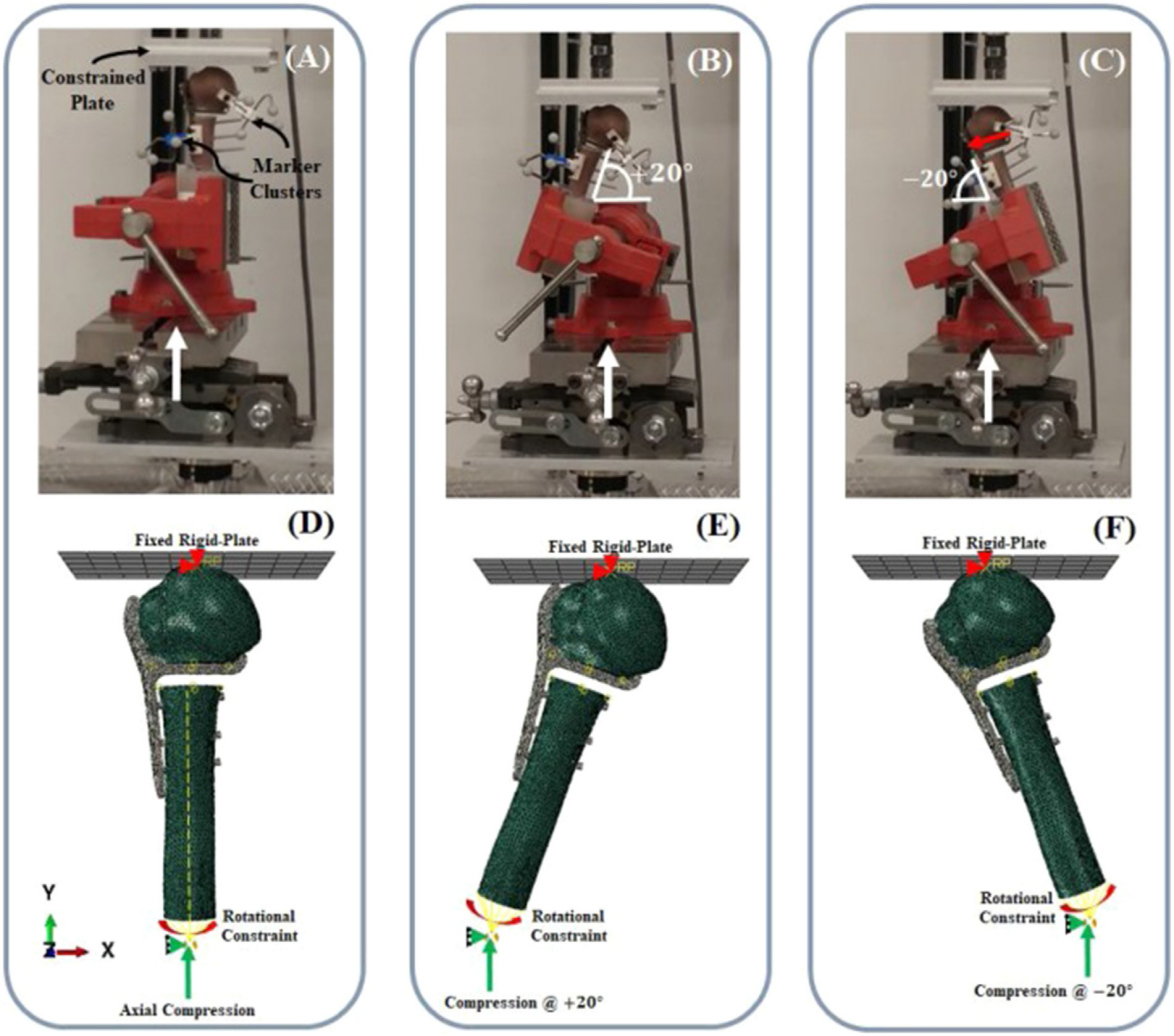
Biomechanical testing of bone plates [67].

Metal AM orthopedics implants may have different tensile, yield, and ultimate strengths than implants made using traditional manufacturing methods. Comparable or marginally greater mechanical strength for metal-assisted metal implants has been reported in certain studies, particularly when using high-performance alloys such as titanium-6 aluminum-4 vanadium (Ti-6Al-4 V). However, the presence of porosity or density variations that are specific to the AM process can also have an impact on the mechanical strength [[64], [65], [66]]. Fig. 6 explains the biomechanical fatigue testing of bone plates fitted with bone models.

**Fig. 7:**
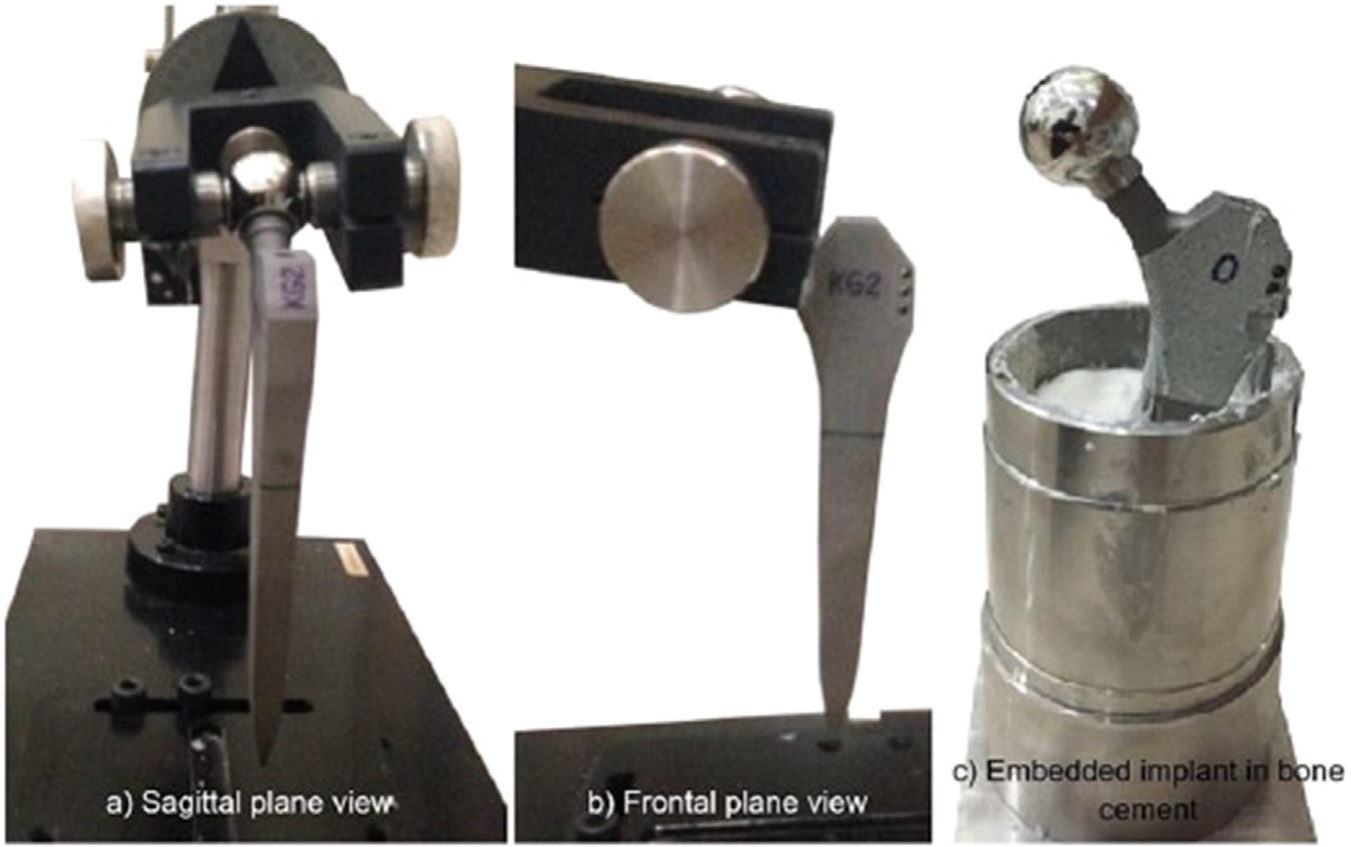
Fatigue test of Hip implant [50].

The fatigue performance of orthopedic implants is crucial, as they are constantly subjected to cyclic loading during daily activities. This repetitive stress demands implants with high durability and resistance to fatigue failure, ensuring long-term reliability and functionality (Fig. 7). When compared to implants that are manufactured conventionally, metal additive manufacturing (AM) implants have demonstrated inconsistent fatigue resistance. While some studies point to similar or better fatigue behavior, others highlight possible issues because of flaws [[68], [69], [70]] or porosity [71].

It is noteworthy that the mechanical characteristics of metal AM orthopedic implants can be significantly influenced by a number of variables; including the particular AM method, design specifications, post-processing procedures, and material selections. Thus, before metal AM implants are used in clinical settings, thorough testing and assessment are essential to guarantee their mechanical integrity and functionality.

#### Stress shielding effect in metal additive manufactured orthopedic implants

Stress shielding is an important biomechanical consideration in orthopaedic implant design, especially in load-bearing applications like hip, knee, and spinal implants. It happens when an implant is significantly stiffer than the surrounding bone, causing the implant to bear the majority of the load, resulting in bone resorption and eventual weakening of the bone structure [72]. Over time, this can result in implant loosening and the need for revision surgery. One of the primary factors contributing to stress shielding is the discrepancy between the modulus of elasticity of metallic implants and that of natural bone [73,74]. While cortical bone has an approximate elastic modulus of 10–30 GPa, commonly used metallic implant materials, such as titanium (110 GPa) and cobalt-chromium (200–230 GPa), have significantly higher elastic moduli. This disparity leads to a stiffer implant, unable to distribute stress effectively to the surrounding bone, thus intensifying the risk of stress shielding [75,76].

Metal additive manufacturing offers unique potential in addressing the stress shielding effect by enabling highly customizable internal structures that can closely mimic the properties of natural bone. Through AM, implants can be designed with optimized porosity and lattice structures to create an effective modulus that better aligns with that of bone tissue. For instance, porous titanium structures with reduced density can achieve a modulus within a range closer to that of cortical bone, facilitating a more balanced load-sharing between the implant and the bone [77]. By fine-tuning the design and porosity of AM-fabricated implants, it is possible to create implants that minimize stress shielding while maintaining mechanical strength and durability. This review examines the impact of metal AM techniques in reducing stress shielding and emphasizes the importance of selecting suitable materials and structures that promote balanced load-sharing between the implant and the bone. Future research in this area is vital, as improved understanding and development of stress-optimized AM implants could significantly enhance patient outcomes and reduce the need for revision surgeries in orthopedic applications [65].

### Applications and case studies highlighting successful implantation

#### Applications

**Fig. 8:**
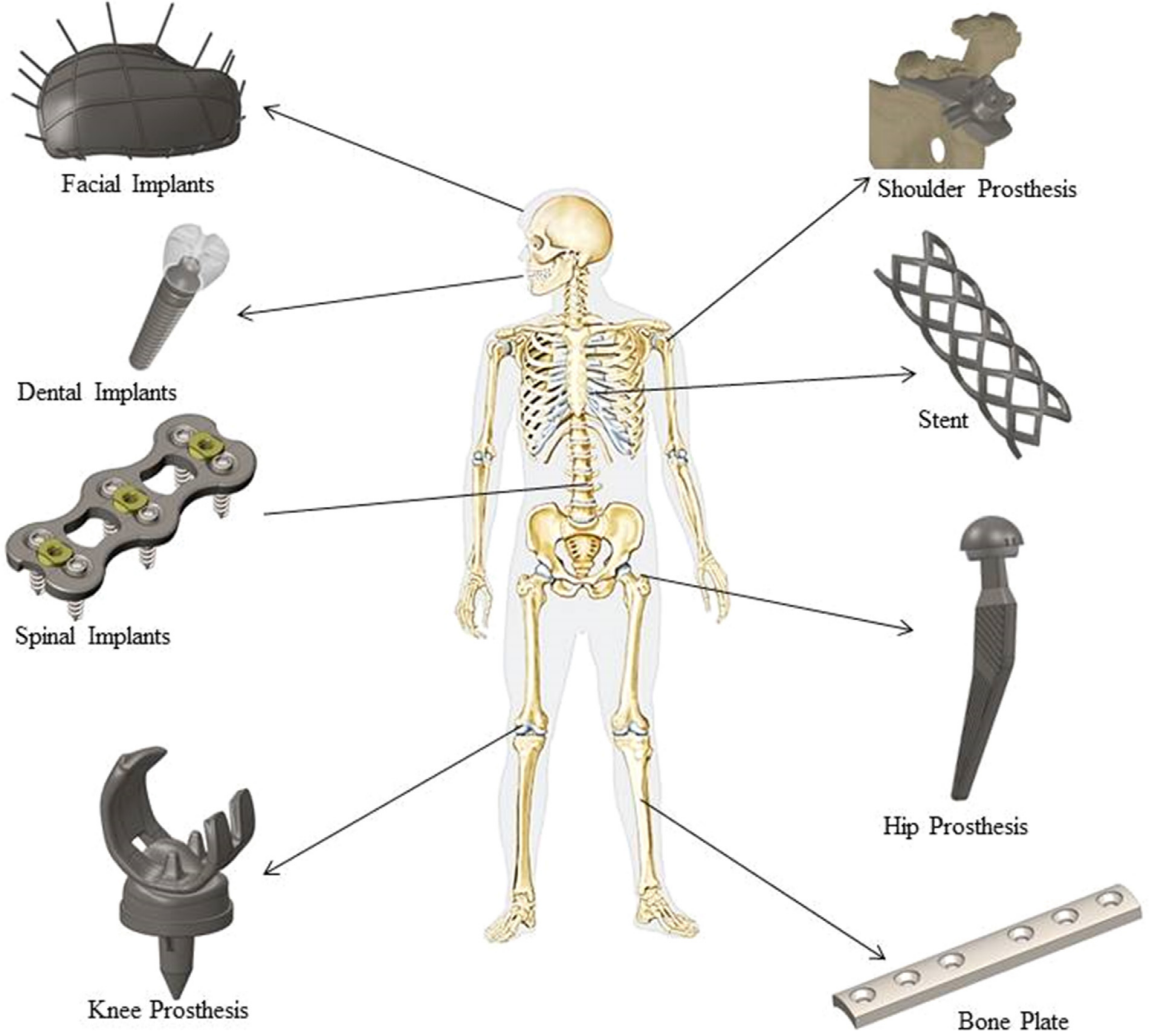
Application of Metal AM in the field of Implants.

It's important to remember that manufacturers and researchers are constantly experimenting with new designs, materials, and uses for metal additive manufacturing (Fig. 8). In orthopedics, where anatomical variations among patients frequently necessitate customized solutions, the ability of metal AM to create complex, patient-specific geometries is especially advantageous. Porous structures can also be incorporated to aid in osseointegration thanks to this technology. The implants in the Fig. 8 were modeled using SOLIDWORKS 2023 modeling Software. The skeleton image in the Fig. 8 is by courtesy of Encyclopædia Britannica [78], Inc., copyright 2015; used with permission.

#### Case studies

One of the top orthopedic companies, DePuySynthes, has been using additive manufacturing to develop orthopedic implants that are customized for each patient. They have used titanium alloy (Ti-6Al-4 V) to make implants, including bone plates, which are specifically shaped to fit the anatomy of each patient. Advanced imaging methods and computer-aided design (CAD) are used in the design process. DePuySynthes' patient-specific implants have been used in a variety of orthopedic procedures, demonstrating the possibility for better patient outcomes, shorter surgical times, and better fit [79,80].

Additive manufacturing has been used by the Australian medical device company Anatomics to create cranial implants customized for each patient. Based on CT scans, they used materials like titanium and polyetherketoneketone (PEKK) to create specialized cranial implants. The implants may have features for improved fixation and are made to precisely fit the anatomy of the patient. Additive manufacturing has been successfully used in cranial surgery by Anatomics, giving doctors customised solutions that improve patient-specific outcomes [81].

Global engineering and scientific technology company Renishaw has experience with orthopaedic applications of additive manufacturing. They have made a contribution to the use of additive manufacturing of titanium in patient-specific jaw reconstruction. In order to reconstruct the patient's mandible, custom titanium implants are made using CT scan data. Better patient recovery has been demonstrated by the potential benefits of using 3D-printed titanium implants to enhance the fit and functionality of jaw reconstruction procedures [82].

In 2019, a patient with a severe cranial defect was successfully treated by physicians at Queen Elizabeth Hospital Birmingham in the UK using 3D printed titanium bone plates. Because of a brain haemorrhage, the patient had a significant portion of his skull removed, and conventional bone plates were considered inappropriate because of the intricate shape of the defect. After a patient's head was scanned, doctors 3D printed a titanium bone plate specifically matched the defect, enabling a successful reconstruction procedure [83].

**Fig. 9:**
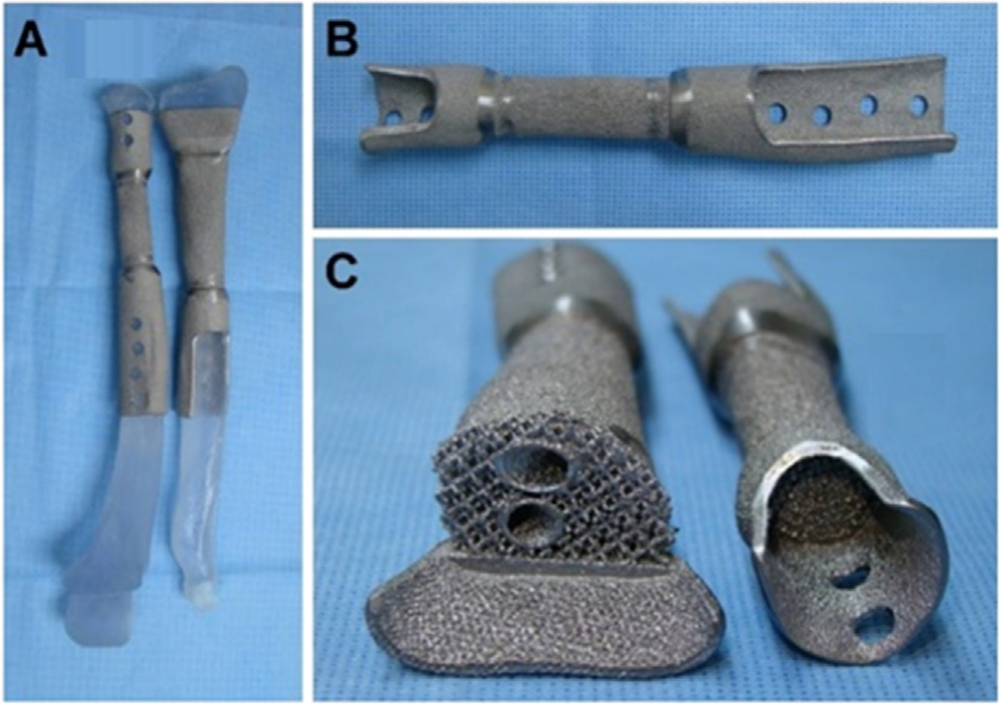
3D printed Ti6Al4 V radius and ulna implant [84].

It may be possible to improve soft tissue integration and osseointegration with an implant made of titanium alloy with the right pore size. There haven't been any reports on the clinical use of these implants on humans, though. Here, the authors describe a case of bone tumour-related limb salvage surgery employing specially made, three-dimensional (3D) printed Ti6Al4 V ulna and radius implants (Fig. 8). The patient had a re-wide excision after presenting with a local recurrence at the proximal junction of the ulna. Following the removal of the recurrent tumour with an ulnar implant, a single forearm bone surgery was carried out utilising a second 3D-printed implant. Histological analysis was used to measure the retrieved implant's host osseointegration and soft tissue integration. At the proximal and distal bone junctions, the implant's overall tissue integration rates were 15.03 % and 45.96 %, respectively. In the proximal and distal bone junctions, respectively, the mesh structure improved bone integration by up to 10.81 % and 8.91 %, respectively. Additionally, in the axial and longitudinal cuts, the implant shaft's soft tissue adhesion rates were 59.50 % and 50.26 %, respectively. Over the entire implant shaft, no space was left empty. Overall, these findings show that the rough-surfaced, 3D-printed Ti6Al4 V titanium alloy implant has a significant capacity for tissue integration [84] (Fig. 9).

### Challenges and future directions in metal AM orthopaedic implant development

It can be difficult to obtain the necessary surface finish straight out of the printing process. The production process is often made more complex and time-consuming by the need for post-processing steps like coating and polishing [[85], [86], [87]]. New materials certification for use in medicine can be a drawn-out and demanding procedure. Even though many different materials are being investigated, it is still difficult to get regulatory approval for them to be used in orthopaedic implants. It is crucial to guarantee uniform quality throughout printed implants. Reliability and reproducibility are difficult to establish because metal AM lacks standardised testing procedures and quality control guidelines [[88], [89], [90]]. Advanced simulation tools are necessary for optimising designs for additive manufacturing, particularly in complex orthopaedic applications. It's still difficult to create reliable models that predict how printed implants will behave mechanically [74,[91], [92], [93], [94]]. The intricate thermal and mechanical processes involved in metal AM may introduce surface irregularities or residual stresses that could affect biocompatibility, even though many of the metal alloys used in AM are naturally biocompatible. Consistent biocompatibility needs to be prioritised [[95], [96], [97]]. Controlled porosity can improve osseointegration, but it can be difficult to strike the right balance between porosity and structural integrity. Long-term success depends on ensuring that implants are properly integrated with the surrounding tissues. It is difficult to increase metal AM implant production while keeping costs low. More focus is required on achieving economies of scale and optimising the production process [[98], [99], [100]].

Continuous research into new alloys and materials, such as composites and nanomaterials, to improve the mechanical characteristics, biocompatibility, and usefulness of orthopaedic implants. developments in multi-material printing technologies to produce implants with gradient structures, which enable the combination of various materials in a single implant to satisfy the biological and mechanical needs of particular geographic areas. Creating control and monitoring systems in real time that evaluate the printed structures' quality during manufacturing [101,102]. Incorporating artificial intelligence (AI) and generative design algorithms to optimize implant designs for patient-specific anatomies and improving the overall efficiency of the design process could improve consistency and decrease the need for extensive post-processing. Establishing standardized testing protocols, quality control procedures, and regulatory frameworks specifically tailored to metal AM for orthopaedic implants could guarantee safety and reliability [103,104]. Improvements in data processing and imaging technologies to enable more precise implants made for individual patients. Large-scale customised solutions might become more possible with mass customization. Investigating and creating bioresorbable metal alloys for implants, especially in fracture fixation, to prevent the need for a second procedure to remove the implant [105,106]. A stronger emphasis on informing engineers and orthopedic surgeons about the potential and constraints of metal additive manufacturing to promote efficient teamwork and practical application of the technology. Future directions in metal additive manufacturing (AM) for orthopedics implants entail tackling present issues via technological innovation, interdisciplinary research, and cooperation between academic institutions, industry, and regulatory agencies. Once these obstacles are addressed, metal additive manufacturing (AM) has the potential to completely transform the orthopedic implant development industry, offering patients more individualized and efficient solutions. [107,108].

In the field of medical implants, a cutting-edge modification technique that shows great promise for future advancements is 3D-printed biofunctional coatings integrated directly during the additive manufacturing process. Unlike traditional post-processing coatings, these biofunctional surfaces are created in-place, allowing for complex, multi-material layering that blends in with the implant's structure. Researchers can use advanced metal 3D printing technologies, such as laser powder bed fusion, to embed biocompatible and antimicrobial agents (such as silver or zinc ions) or growth factors directly into the implant surface layers [109,110]. This approach not only improves biointegration but also provides continuous, localized antimicrobial activity, lowering the risk of infection without the need for systemic antibiotics. Furthermore, by incorporating graded porosity and specific surface textures, implants can achieve modulus matching with the surrounding bone, effectively reducing the stress-shielding effect that frequently results in bone resorption and implant failure. Looking ahead, this in-situ functionalization technique could be further developed to incorporate custom-tailored drugs, peptides, or even stem cell niches that actively promote healing, representing a future in which implants are highly adaptable, patient-specific, and capable of actively supporting long-term biological function and health [111,112].

## Conclusion

The fascinating world of metal additive manufacturing (AM) in orthopedic implant development has been explored in this review paper, which provides a thorough summary of the major discoveries and developments in this emerging field. The transformative potential of metal additive manufacturing (AM) has been unveiled through an examination of the challenges encountered and the anticipated future directions of this technology, in conjunction with an exploration of various metals and alloys. The body of knowledge highlights how important metal AM is to the advancement of orthopedic implant technology. Among the advantages of metal additive manufacturing (AM) are their capacities to customize implants to each patient's unique anatomy, investigate cutting-edge materials, and incorporate complex designs that are impractical for conventional manufacturing techniques. These developments could improve implant functionality, maximize osseointegration, and eventually lead to better patient outcomes. At the forefront of this game-changing technology, we must acknowledge the ongoing challenges that lie ahead, such as the requirement for standardized testing, regulatory approval, and ongoing advancements in materials science and design optimization. However, these difficulties ought to be seen as chances for additional study and cooperation. Because metal AM has such great potential, it will require multidisciplinary efforts from researchers, physicians, engineers, and regulatory agencies to continue exploring it. This review urges a concentrated effort to advance metal AM technology in the creation of orthopedic implants. To guarantee the security, performance, and dependability of printed implants, cooperative projects should focus on improving the production procedures, broadening the range of materials, and developing standardized procedures. Furthermore, a greater comprehension of the potential and constraints of metal AM will be facilitated by the demand for more education and training in the engineering and medical fields. There exists a great potential for advancement and innovation as we explore the limits of metal additive manufacturing in the development of orthopedic implants. By means of sustained investigation, cooperation, and a resolute dedication to surmounting obstacles, metal AM is well-positioned to fundamentally alter the terrain of orthopedic treatment, providing tailored and optimal remedies for patients globally.

## Ethics statements

The paper reflects the authors' own research and analysis in a truthful and complete manner.

## CRediT authorship contribution statement

**Senthil Maharaj Kennedy:** Conceptualization, Methodology, Software, Writing – original draft. **Vasanthanathan A:** Supervision, Writing – review & editing. **Amudhan K:** Visualization, Data curation, Investigation.

## Declaration of competing interest

The authors declare that they have no known competing financial interests or personal relationships that could have appeared to influence the work reported in this paper.
